# Evaluation of the Effectiveness of Five Odor Reducing Agents for Sewer System Odors Using an On-Line Total Reduced Sulfur Analyzer

**DOI:** 10.3390/s121216892

**Published:** 2012-12-07

**Authors:** Il Choi, Hyunjoo Lee, Joungdu Shin, Hyunook Kim

**Affiliations:** 1 Department of Energy and Environmental System Engineering, University of Seoul, 90 Jeonnong-dong, Dongdaemun-gu, Seoul 130-743, Korea; E-Mails: il_choi@hangulmail.com (I.C.); hyunjoo901@naver.com (H.L.); 2 Department of Climate Change & Ecology, National Academy of Agricultural Science, RDA, Suin-ro, 126th, Gwonseon-gu Suwon Gyeonggi-do, Suwon 441-707, Korea; E-Mail: jdshin@rda.go.kr

**Keywords:** sewer odor, on-line TRS analyzer, odor-reducing agent, NaOCl

## Abstract

Sewer odors have been a concern to citizens of the Metropolitan Seoul region, which has installed combined sewer systems (CSSs) in 86% of its area. Although a variety of odorants are released from sewers, volatile sulfur compounds (VSCs) have been recognized as major ones. A number of technologies have been proposed to monitor or control odors from sewers. One of the most popular strategies adopted for the control of sewage odor is by applying a commercial odor-reducing agent into the sewer. In this study, the effectiveness of five different commercial odor-reducing agents (*i.e.*, an odor masking agent, an alkaline solution, two microbial agents, and a chemical oxidant) was evaluated by continuously monitoring VSCs released from the sewer with an on-line total reduced sulfur (TRS) analyzer before and after each agent was sprayed into CSSs at five different locations of the city. In short, when the effectiveness of odor treatment was tested in the sewer system using five commercial odor reducing treatments, only the chemical oxidant was good enough to reduce the odor in terms of TRS levels measured before and after the application (*p* < 0.01).

## 1. Introduction

The urban sewer system is an important infrastructure supporting the safety and comfort of citizens. The role of the sewer system becomes even more important in a rapidly growing city. In an old city like Metropolitan Seoul, a combined sewer system (CSS) which collects sewage and storm water together is common; in fact, about 86% of the Metropolitan Seoul area is covered by a CSS. One of the major issues associated with the CSS is the sewage odor emitted from sewer pipes, especially through street inlets or manholes [1].

The odorous gas from sewer systems contains volatile sulfur compounds (VSCs), nitrogen compounds and fatty acids. Ammonia, a major odorant from the sewer system, is a byproduct generated from the biodegradation of nitrogen-containing organic compounds, for example H_2_N-CO-NH_2_ (urea) → NH_3_ + CO_2_. The urea concentration of sewage is high because urine excreted by humans contains about 25 g·L^−1^ urea [2].

Biological and chemical mechanisms for the generation or decomposition of some sulfur compounds in the sewer under aerobic and anaerobic conditions are well-documented. The major reactions involving sulfur compounds in sewage are: (1) the reduction of sulfate to sulfide by sulfur reducing bacteria; (2) the decomposition of amino acids containing sulfur; (3) the methylation of methyl mercaptan (CH_3_SH) by H_2_S and (4) dimethyl sulfide (DMS) generation via oxidation of CH_3_SH [3–7].

The VSCs including H_2_S are causing not only bad odors [8,9], but also the corrosion of the concrete sewer pipes [7]. Sulfate ion as a precursor of H_2_S flows into sewers, because residential sewage contains significant amount of sulfate salts from the use of detergents. As the slime layer on the wall of sewer pipes increases and the sludge deposition increases on the bottom of pipes, more sulfate salts are biologically transformed to H_2_S; the production and emission of H_2_S in the sewer are influenced by the retention time of wastewater, and other factors such as water depth, temperature, BOD and pH [10]. In fact, H_2_S is the source for the sulfuric acid in the slime layer on the sewer pipes, which accelerates the corrosion of concrete pipe.

Among a number of VSCs, H_2_S has been pointed out as the most important compound contributing to odor complaints from the sewer system [3,10,11]. In fact, a good correlation between odor concentration (standard odor units m^−3^) and H_2_S concentration (mg·L^−1^) of wastewater had been found and the latter has even been utilized to estimate the odor concentration [10]. Therefore, the monitoring and control of VSCs including H_2_S from the sewer could be the most important task to reduce public odor complaints about the sewer system.

Recently, a few researchers have successfully applied on-line total reduced sulfur (TRS) analyzers for the monitoring of sulfur emissions from sewage, sludge, and unit processes of a wastewater treatment plant (WWTP) [12,13]. For example, in the study performed at the Blue Plains WWTP in the District of Columbia (USA) [12], a TRS analyzer was successfully utilized to monitor VSCs from bio-solids handling processes. The Ontario Ministry of the Environment (Canada) regulates the ambient odor quality near a WWTP, using the data obtained with on-line TRS and SO_2_ analyzers [14]. In the case of a WWTP near Nearys Lagoon in the City of Santa Cruz (CA, USA) [15], the TRS is monitored at the end of the force main near the WWTP of the city for predicting potential odor release from the plant. Several WWTPs in Korea [16] also monitor VSCs from their unit processes using on-line TRS analyzers.

A few strategies have been proposed to reduce the odor emission from the sewers in Korea. They include the modification of street inlets, increasing the treatment efficiency of sewage disposal tanks, the reduction of eddy flow occurring when the outflow pump of a sewage disposal tank is on, and spraying (or injecting) an odor-reducing agent into sewer pipes (or sewage). Especially, the last one is often selected by local governments in South Korea, because it is easy to implement, and a number of odor reducing agents have been commercialized in South Korea for this purpose. However, the evaluation of their efficiency has not been performed objectively and systemically. Therefore, local governments are often confused when selecting a proper odor-reducing agent.

Conventional evaluation of the effectiveness of a technique to control odors from sewer systems involves collecting gas samples for instrumental analysis before and after the control technique is applied. In fact, since odors are generated instantaneously from the sewer, intermittent gas sampling and analysis often fail to determine the effectiveness of an odor control technique of interest. In order to properly monitor and control the intermittently-occurring odors, a strategy to continuously monitor odor emission is required.

The objective of the current study was to objectively evaluate the efficiency of five commercial odor-reducing agents on sewer odor. Therefore, the agents were sprayed into the sewer in five different commercial districts; one agent for one district. VSC emissions from the sewer were continuously monitored using a TRS analyzer before and after each agent was sprayed into the sewer pipes at each study site. Based on the VSC emission profiles observed at each site, the efficiency of the odor-reducing agent applied to the site was evaluated. In addition, gas samples were also collected from each site before and after an agent applied, and analyzed for individual odorants.

## 2. Experimental Section

### 2.1. Odor Reducing Agents Evaluated in This Study

In this study, a total of five odor-reducing agents were evaluated for their effectiveness in controlling sewer odors; four are commercially-marketed odor-reducing agents, and one is a common chemical oxidant [17]. They are a masking agent made of phytoncide (Agent-A), a water-based agent with natural minerals (Agent-B), a biological agent in the dormancy state (Agent-C), a gel-type agent made from natural plants (Agent-D), and NaOCl (Agent-E). The characteristics, working principle, and spraying method of each agent are summarized in Table 1. The manufacturers of the commercial odor-reducing agents A, B, C, and D did not disclose detailed information on the ingredients of their agents. The application rate and method for each odor-reducing agent were determined following the manufacturers’ directions.

### 2.2. Study Locations

**Figure 1.**
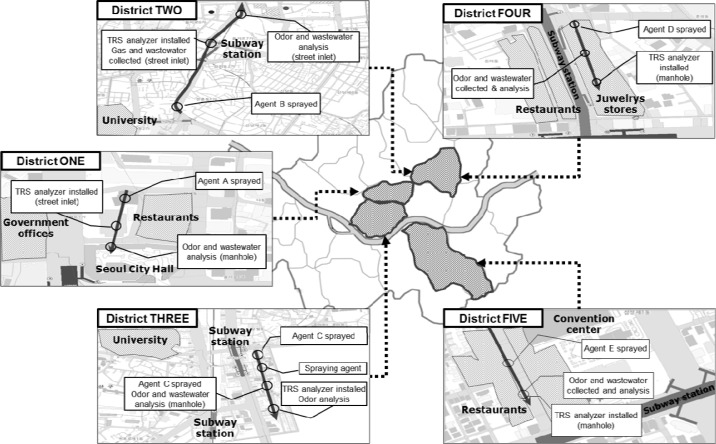
Study sites in Seoul.

Experiments were performed at five commercial areas in Seoul, South Korea (Figure 1). These particular study sites have been the target of the odor complaints because there are many fancy shops and luxury restaurants attracting large floating populations. Therefore, for these sites, five odor reducing agents were applied and their effectiveness was evaluated; one agent was applied to one site.

The odorous sulfur gases, as an indicator for odor sensation from each site were continuously monitored using an on-line TRS analyzer (M102E Teledyne Instrument, San Diego, CA, USA) installed at a manhole of each site. Each odor-reducing agent was sprayed at a point of 300–500 m upstream from the location where the TRS analyzer was installed. The followings are brief descriptions of each study site:

District-ONE is located near the Seoul Metropolitan City Hall. In the district, there are many fancy restaurants and coffee houses as well as government offices. Therefore, this district is busy at lunch and dinner times, when large amounts of wastewater are discharged.

District-TWO is located along a 2 km-long side road where 32 commercial high rise buildings and 28,000 shops are located. Since this district is located near Cheonggye-cheon Stream (one of the most popular tourist destinations in Seoul), it is always crowded, *i.e.*, several million people visit the location every day. Therefore, a relatively large amount of wastewater is always discharged into the sewer.

District-THREE is a market place formed around a subway station and a woman’s university with numerous fashionable boutiques for young women and food/drink establishments. The daily averages of the floating population in the district are about 60,000–70,000 on weekdays and about 20,000 on weekends. The floating population peaks at 3–6 p.m. and this area is always busy till late at night. As a result, the amount of wastewater increases in the evening.

District-FOUR is located on a side street where small shops and theaters are located. The daily floating population of the area is about 180,000. Young people in particular gather in the district from the evening till the dawn.

District-FIVE is located near a convention center and there are many restaurants and offices as well as luxury hotels. Therefore, this district is busy at lunch and dinner times, when a large amount of wastewater is discharged.

### 2.3. Evaluation of Odors from Sewer Pipes

#### 2.3.1. On-Line TRS Analyzer

An on-line TRS analyzer was installed at a manhole in each study site. The headspace gas of each manhole was collected once every five min and was fed into the TRS analyzer. The TRS analyzer thermally oxidizes reduced sulfur compounds in a sample gas to SO_2_, which is subsequently quantified with an optical sensor [18].

**Figure 2.**
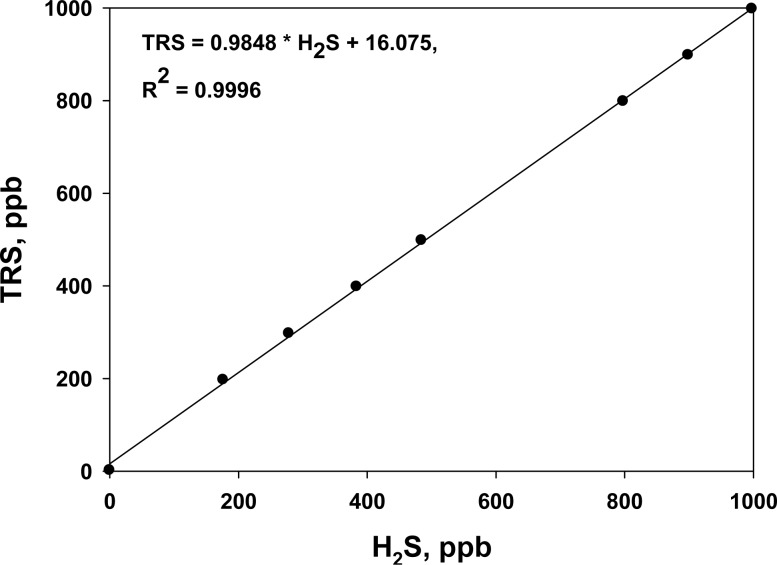
Example calibration curve for on-line TRS analyzer against standard H_2_S gas.

Before each on-line TRS analyzer was installed, it was calibrated with standard H_2_S gas. In addition, the instrument was regularly maintained to ensure accurate measurements. The R^2^ of the calibration curves was always higher than 0.9996 in the range 0 to 1,000 ppb (Figure 2). The TRS analyzers were operated for a week before and after the treatment at each site.

**Figure 3.**
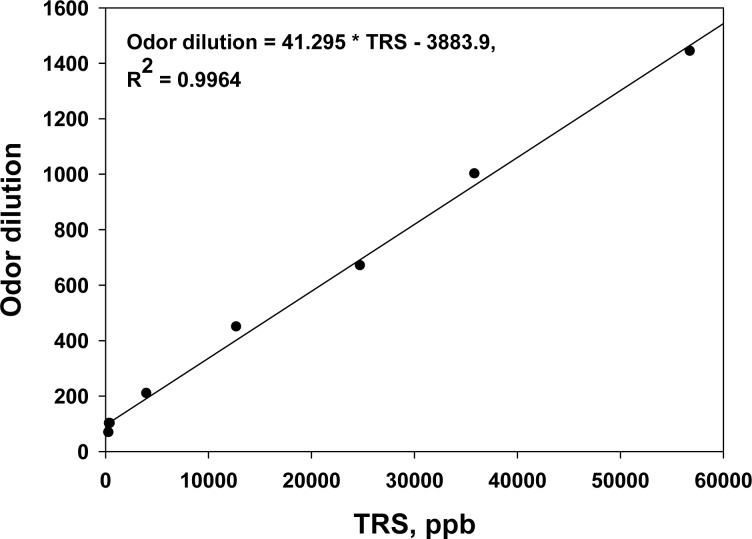
Odor dilution *vs.* TRS concentration from the sewers in the study sites.

In order to confirm the major odorous compounds from sewer systems, two or three gas samples were collected from the inside of a manhole located in each study sites, except District-FIVE, and were analyzed 1–2 months before the deodorant evaluation study was initiated. In fact, it was found that sulfur compounds were the major odorants. The TRS concentration measured by the TRS analyzer on-site and odor dilution of the samples collected from the sites showed a good linear correlation (Figure 3). Therefore, it was confirmed that the on-line TRS analyzer could be used as a tool to monitor the odor emissions from sewer systems and to evaluate the efficiency of any odor reducing technology.

#### 2.3.2. Measurement of Odor and Individual Odorants

In general, sampling and analysis of odorous gases were performed following the Korean Odor Measurement and Analysis Methods [19,20]. Brief explanations of the sample collection and analysis methods are provided below.

##### Collection, Transportation and Storage of Samples

The gas samples were taken from the monitoring site of each district (e.g., from a street inlet or a manhole located on 300–500 m downstream from the agent-spraying point) at three different times before and after the treatment. The gas samples were collected for 5 min with a diaphragm pump regulated by a flow meter and operating at 4 L·min^−1^ using a 20 L Tedlar bag. Tedlar bags were cleaned three times with high purity nitrogen before use and the pump and the tubes were also cleaned by flowing high purity nitrogen through them for 3 min. After collection, samples were stored in light resistant containers, and were sent to a third party operating a certified laboratory for determining odor dilution, and to our laboratory for instrumental analysis of individual odorants. All the samples were analyzed within a day after collection.

##### Determination of Odor Dilution

Odors of collected gas samples were determined by a third party panel. The samples for the panel evaluation were mixed with odor-free air with the dilution factor of 3, 10, and 30 times and so on using an auto-dilution unit. The odor panel was composed of five males over 19 years old. All of them had passed a screening test specially designed for panel candidates.

##### Analysis of Individual Odorants

A set of gas samples were collected and analyzed for individual odorants (*i.e.*, NH_3_, H_2_S, CH_3_SH, DMS, and DMDS) following the Korean Odor Measurement and Analysis Methods [19]. For NH_3_ analysis, the gas in the headspace of each sewer was absorbed by an acid solution in a bottle; the gas was passed through the acid solution at 10 L·min^−1^ for 5 min. The concentration of the absorbed NH_3_ was quantified following the standard methods. Sulfur compounds (*i.e.*, CH_3_SH, H_2_S, DMS, and DMDS) in a gas sample collected by a Tedlar bag were analyzed by a cold concentrated-capillary column GC-FPD method. In addition, the effect of odor reducing agents on the water quality of the wastewater in the sewer at each site was also evaluated by collecting water samples before and after the treatment at each site, and by measuring water temperature, pH, BOD, SS, and NH_3_.

## 3. Results and Discussion

### 3.1. Effectiveness of Agent-A in Control of Odors from Sewer

**Figure 4.**
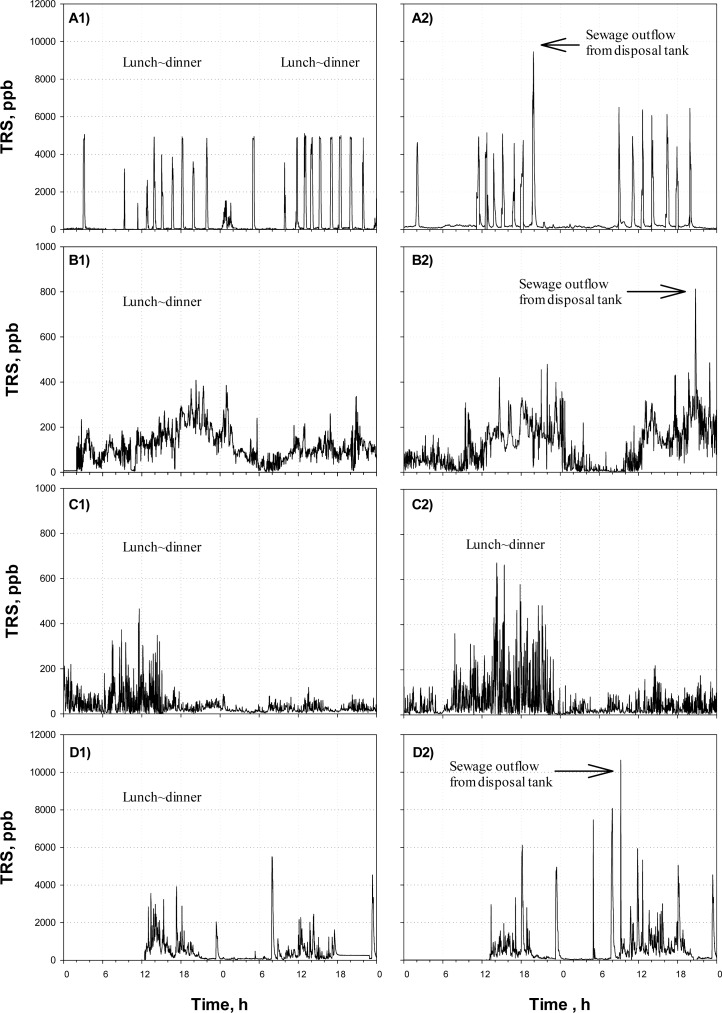
Time profiles of TRS measured at Districts-ONE–FOUR before and after application of Agents-(A–D).

Figure 4(A) shows the TRS profiles monitored by the on-line TRS analyzer before (A1) and after (A2) Agent-A was applied at District-ONE.

Since a few governmental office buildings are located in the area along with many restaurants and coffee shops, large amounts of wastewater are intermittently discharged from the noon to evening. The wastewater discharge is also identified by the on-line TRS analyzer. From Figure 4(A), intermittent releases of large amount of sulfur compounds were observed; especially the peaks often appeared from lunch time to the evening. On the other hand, low concentrations of TRS were detected from dawn till noon. The times when the TRS peaks were observed were identical to the pumping times for outflows from the septic tanks in the large buildings along the street, indicating that the odor emission from the sewer was mainly due to the activity of the commercial areas. It was found that there was little difference between the TRS profiles before and after the treatment were compared. The daily average TRS concentrations of the site before and after the Agent-A was applied were almost the same; *i.e.*, 370 ppb and 410 ppb for before and after the treatment, respectively.

At the study site of the District-ONE, the gas samples were collected before and after the agent applied, and analyzed both for odor and individual odorants. The samples were collected during the day times (13:00–15:00). The odorous gas samples were collected three times at intervals of 20–40 min. In general, there was not a statistically significant difference in the odor dilutions obtained from the panel evaluation for the gas samples collected before and after the application of the Agent-A (*p* = 0.91). Although gas samples for measuring odor dilution were collected when two odor panels were noticed odor emission, still large deviations could be observed from the study (Table 2). However, it should be noted that olfactory measurement can produce large deviations, especially at lower odor dilutions [11].

In the instrumental analysis for individual sulfur odorants, H_2_S and CH_3_SH concentrations were higher than DMS or DMDS both before and after the agent treatment. The average NH_3_ concentrations before and after the treatment of the Agent-A were 0.6 ppm, respectively. Considering the published odor threshold for NH_3_ (*i.e.*, 4.1–37 ppm, [21]), contribution of NH_3_ to odor sensation might not be significant at the site.

Since a masking agent like Agent-A does not degrade nor transform odorants from sewer systems, but it literally covers the sewer odor with its strong and relatively pleasant odor [22,23], it was not expected that degradation of odorants would happen. From the fact that the odor dilution values measured before and after the treatment were not much different and the odor characters were also similar, it was assumed that the masking agent failed to cover the sewer odor enough.

The results from the analysis of wastewater collected from the site before and after the treatment of the Agent-A showed little difference in water qualities, except BOD (Table 3). However, we do not think that the BOD increase was due to the added odor-reducing agent, since the amount of the agent applied to the site should be much smaller than that of wastewater generated in the district (Table 1).

### 3.2. Effectiveness of Agent-B in Control of Odors from Sewer

Figure 4(B) shows the TRS profiles obtained at the District-TWO before (B1) and after (B2) the application of the Agent-B. In this area, not much odor-causing gas was released. The TRS concentration was always within 0–500 ppb with a relatively high concentration during the day.

Regarding the effectiveness of the Agent-B, the agent did not result in any difference in the time profiles of TRS concentration observed before and after the treatment (Figure 4(B)). The daily average TRS values were not different, either (*p* = 0.34); the daily average was 100 ppb before the agent was applied, while it was 140 ppb after the agent with applied.

The instrumental analysis performed with the gas samples collected at the District-TWO showed a similar result to that of the samples collected from District-ONE. The odor dilution and the concentrations of individual sulfur compounds of gas samples collected before and after the Agent-B treatment were not much different except for CH_3_SH (Table 2). The result of the analysis with the wastewater samples collected before and after the agent treatment did not show much difference (Table 3).

In the case of District-TWO, in fact, the sprayed amount of the solution (*i.e.*, Agent-B which is alkaline solution) was relatively large, compared to other agents applied in this study. However, it could not effectively prevent the release of H_2_S from the sewage; as shown in Figure 4(B), the profiles of TRS concentration measured before and after the agent were almost identical. In addition, the sewage pH did not change much. From these findings, it was assumed that the dose of Agent-B applied in District-TWO was not enough to result in any changes of sewage characteristics. Therefore, it was concluded that the application of the odor-reducing agent (*i.e.*, Agent-B) were not effective.

### 3.3. Effectiveness of Agent-C in Control of Odor from Sewer

Figure 4(C) shows the time profiles of TRS concentration monitored at the District-THREE before (C1) and after (C2) application of the Agent-C. The site was characterized by the large difference in TRS emissions between day and night. From Figure 4(C) the TRS observed at night was far lower than those observed during the daytime. It was because the floating population of the weekdays was 3–4 times higher than that of weekend. After Agent-C was applied, the weekly average concentration of TRS was relatively lower than those of Districts-ONE and -TWO.

Peaks in the TRS profiles could be observed after noon, while the detected TRS level was low from midnight till the noon (Figure 4(C)). As the cases of the Agents-A and -B, the time profiles of TRS emission observed before and after the odor-reducing agent was applied did not show statistically much difference, even though the average of TRS emissions recorded after the Agent-C treatment was lower than that recorded before the treatment; the daily average TRS concentrations measured before and after the treatment were 65 ppb and 40 ppb, respectively.

The odor dilution and the concentration of individual odorants of the samples collected at the manhole where the on-line TRS analyzer was installed in District-THREE showed similar results to those obtained from Districts-ONE and -TWO (Table 2); the odor quality of the gas samples was not improved by the application of the odor-reducing agent (*i.e.*, Agent-C). In fact, odor dilution after the agent treatment was higher than those observed before the treatment (*p* < 0.05). The discrepancy between the odor dilution and the average TRS values was attributed to the possibility that more odorous gases were collected when the grab-sampling event happened after the agent treatment. Other individual odorants and H_2_S were also detected at higher levels after the agent had been applied.

The result from the analysis of wastewater samples collected at the District-THREE revealed the Agent-C did not have any effect on the water quality of the sewage in the sewer, either. Although higher BOD and SS levels could be observed after the agent treatment, we do not believe it was because of the addition of the agent.

### 3.4. Effectiveness of Agent-D in Control of Odors from Sewer

Figure 4(D) shows the time profiles of TRS concentration observed in District-FOUR, before (D1) and after (D2) Agent-D was applied. Since the area is occupied with a number of small-to-medium size shops and jewelers, the floating population increases at lunch and dinner times on weekdays, particularly from 12–19 o’clock. The changes of the floating population affected the amount of wastewater produced in the district.

In general, the TRS concentration was high from the noon to the evening, with several intermittent high peaks (Figure 4(D)). In fact, the shops and jewelers in the district were open late in the morning at 9:00 to 11:00; so low TRS emission could be observed in the early morning. After the shops were closed after the evening, the TRS emission decreased below 100 ppb till the morning of the next day. The high TRS concentration peak observed at around 22–23 o’clock was found to be due to the pumping of wastewater from the septic tank into the sewer in a nearby building.

Regarding the effect of the Agent-D on the TRS emissions from the District-FOUR, the agent did not the one observed before. The average TRS value was also higher after the treatment; the average TRS was 210 ppb before the agent treatment but it increased to 415 ppb when measured after the treatment.

The odor dilution and the concentration of individual odorants of the samples collected from a manhole in the District-FOUR showed the similar result as those obtained from the Districts-ONE, -TWO, and -THREE. H_2_S and mercaptans were found to be the major odorants contributing to the odor sensation of the district (Table 2). The analyses of wastewater performed after the Agent-D treatment confirmed that the addition of the agent did not have any effect on the water quality of wastewater in the sewer, either (Table 3).

Microbial organisms have been extensively applied to treat municipal wastewater, and livestock wastes, since they can effectively degrade organic matter in wastewater [24]. Microorganisms live naturally in sewer systems and they digest solids and breakdown various components. Based on the assumption that the microbes can degrade odorants in wastewater or sewage, microbial additives have been applied to sewer systems and waste treatment facilities. It is true that little information is available regarding the effectiveness of microbial additives in reducing odors. In their recent study, Rahman *et al.*[25] evaluated the efficacy of a commercial microbial additive in reducing odors from a swine manure pit, and found no difference in terms of odor, ammonia, and H_2_S between treated and untreated pits. However, Reimers *et al.*[26] observed effective reduction of H_2_S emission in their study where a microbial agent was applied to a collection system in front of a wastewater treatment plant at a rate of 1 L additive solution per 1,000 m^3^ wastewater.

Nonetheless, in our study both microbial additives applied to District-THREE and District-FOUR did not show any reduction in odor release, possibly because the dose was not enough to reduce the sewer odor. In addition, the application should last for a long period of time. Reimers *et al.*[26] had applied the additive for more than two years in order to reduce hydrogen level from 3.5 ppm to below 1 ppm. However, in our case, Agent-C and Agent-D had been applied to sewer systems only for a month before we evaluated their effectiveness in terms of odor, and H_2_S reduction.

### 3.5. Effectiveness of Agent-E in Control of Odors from Sewer

**Figure 5.**
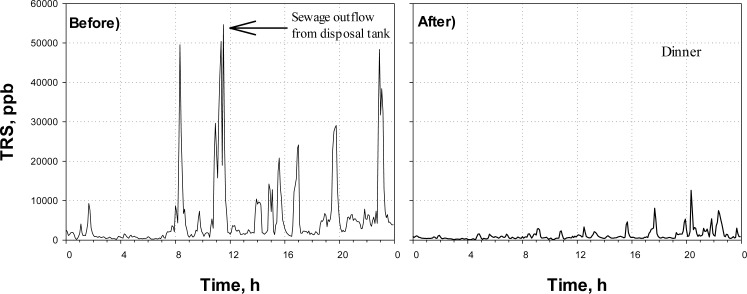
Time profiles of TRS measured at District-FIVE before and after application of Agent-E.

Figure 5 shows the time profiles of the TRS concentration obtained at the District-FIVE before and after the Agent-E (*i.e.*, NaOCl) was applied. This district is occupied by office-buildings, restaurants, hotels, and a convention center. Since the district is heavily populated, wastewater is continuously generated and flows into sewer. Therefore, sewage odor almost always could be sensed in the district.

From (Figure 5-Before), the TRS concentration observed in a manhole of the study site was much higher than those observed other sites. The TRS concentration was often over 20 ppm during a day before the Agent-E was applied. In fact, the District-FIVE is notorious with the sewer odor, which has been a target of the public complaints.

After the NaOCl was injected (Figure 5-After), the TRS concentration was not over 10 ppm. In addition, the time when TRS emission was high reduced significantly after the chemical oxidant was injected; the total time when the TRS was over 5 ppm in the day in Figure 5 was 395 min before the oxidant was applied, while it decreased to 60 min after the agent was applied.

We collected gas samples at a manhole near the site where the on-line TRS analyzer was installed before and after the oxidant was applied. The odor dilution and individual odor-causing compounds of the gas samples were analyzed (Table 2). After inserting the oxidant into the sewer of the District, the odor D/T was significantly reduced from over 6,000 to around 300 (*p* < 0.05), while H_2_S concentration was reduced from 25 ppm to 2 ppm (*p* < 0.01). In fact, OCl^−^ can be consumed to oxidize sulfur compounds (e.g., mercaptans, disulfides, *etc.*) to SO_4_^2−^; for example, HS^−^ in sewage can be oxidized by OCl^−^ (Equation (1)). Since hydrogen sulfide is the main odorant in a sewer system, transformation of sulfide by an oxidant to sulfate ion definitely contributed to reduction of odor emission in the current study:

(1) $${\text{HS}}^{-}+{4\text{OCl}}^{-}\to {\text{SO}}_{4}^{2-}+{4\text{Cl}}^{-}+{\text{H}}^{+}$$

In general, microbial activities are responsible for the malodor emission from the anaerobic sewer systems. Another benefit of hypochlorite addition is disinfecting odor generating anaerobic microorganisms such as sulfate reducing bacteria. The disinfection effect of OCl^−^ solution also must have contributed to the control of hydrogen sulfide generation from the sewer system [27].

The water quality of the wastewater seemed to be affected slightly (Table 3); the BOD and NH_4_^+^ concentrations of the wastewater decreased. However, it was not confirmed the reduction of BOD and NH_4_^+^ was due to the addition of the oxidant. Nonetheless, it was concluded that application of the NaOCl was effective.

## 4. Conclusions

In this study, odor removal efficiencies of five different odor-reducing agents were evaluated using on-line TRS analyzers. Since odor sensation is an instant event, one-time grab sampling and analysis of a gas sample often fail to explain how odors are emitted. Therefore, total amount of VSCs (as an indicator for odor sensation) emitted from the sewer before and after each odor-reducing agent was applied were monitored using the TRS analyzer. In fact, using on-line TRS analyzers, we could successfully monitor the overall odor emission pattern at each sewer site and could objectively evaluate the efficiency of each odor-reducing agent.

Using the on-line TRS analyzers, we could find that odor emissions from sewer systems were highly related with the wastewater outflow from septic tanks installed in the underground of buildings in the study sites. Whenever the pumps were turned on to pour wastewater out of the septic tanks, peaks on the TRS profiles could be observed.

As for the effectiveness of the deodorants under study, only the chemical oxidant (*i.e.*, NaOCl, Agent-E) was effective in reducing or controlling odor emissions from the sewers. The effectiveness of the oxidant injection was confirmed by comparing the TRS profiles observed before and after the oxidant was applied. It was also confirmed by the odor dilutions measured for the gas samples collected from the site (District-Five); the dilution-to-threshold (D/T) measured before and after the oxidant was applied were 6,000 and 300, respectively.

Unfortunately, none of the other odor-reducing agents (*i.e.*, Agents-A through-D) improved the odor quality of gas emissions from the sewers at each study site. We could not find statistically significant reductions of the odor emissions in Districts-ONE through -FOUR. The failure of the commercial deodorants in reducing the odor emission from the districts was attributed to the possibility that the amounts of each agent applied in this study were insufficient, even though we followed the application directions in each case.

In conclusion, when an odor reducing agent is selected to reduce or control sewer odors, a systematic evaluation of each candidate in a full scale should be performed. In addition, utilizing an on-line measurement tool like the TRS analyzer used in this study will be beneficial in the evaluation and selection of the right deodorant.

## Figures and Tables

**Table 1.** Characteristics of odor reducing agent used in study.

**Contents**	**A**	**B**	**C**	**D**	**E**
Characteristic and specification	Phytoncide extracted from woody plants Alkaline	Alkaline solution with Ca, K, Mg, Na, Si, *etc.*	Munizyme (US product). Bacta-Pur (Canadian product) Activated microorganism	Biomass mixed with natural ingredients	0.2% NaOCl
Working principle	Emission of forest smell to mask bad odor	Increasing wastewater pH to prevent H_2_S or fatty acids from releasing from sewer	Biodegradation of odorants	Biodegradation of odorants in sewage	Oxidation of odorants
Method of spraying	Spray into the sewer by a dispenser attached to the side wall under the manhole cover	Sporadic spray of the solution	Bacta-Pur: spray via a dosing machine Munizyme: spray via a dosing machine	Spray in the headspace of sewer pipes	Spray in the headspace of the sewers
Quantity sprayed	76 mL·d^−1^ per manhole; the night time of 8 h excluded	16.7 L·h^−1^	Munizyme: 42 mL·h^−1^ Bacta-Pur: 42 mL·h^−1^	50 mL·h^−1^	3 L·h^−1^

**Table 2.** Results of odor and odorants measurement at Districts A, B, C, D and E.

**Sites**	**Before or after agent application**	**Dilution to threshold, D/T**	**NH_3,_ ppm**	**Sulfur compounds**			
				**H_2_S, ppb**	**CH_3_SH, ppb**	**DMS, ppb**	**DMDS, ppb**
ONE	Before	370 (550) *	0.6 (0.1)	7,500 (13,000)	185 (310)	8 (11)	0.1 (0.1)
	After	410 (290)	0.6 (0.3)	5,000 (4,900)	200 (200)	4 (4)	1.5 (2.4)
TWO	Before	90 (20)	0.3 (0.2)	150 (19)	25 (4)	3 (0.6)	0.5 (0.1)
	After	100 (50)	0.7 (0.1)	175 (125)	50 (30)	4 (2.5)	0.8 (0.5)
THREE	Before	20 (6)	0.2 (0.1)	20 (32)	2.2 (4)	0.1 (0.2)	0.1 (0.1)
	After	40 (8)	0.4 (0.3)	65 (30)	7.3 (4)	0.5 (0.8)	0.1 (0.1)
FOUR	Before	60 (24)	0.5 (0.2)	360 (530)	7 (10)	0.6 (1.0)	ND ^**^ (-)
	After	40 (24)	1.0 (0.1)	220 (330)	7 (8)	1.5 (0.7)	0.1 (0.1)
FIVE	Before	6,000 (4,600)	6 (0.1)	25,000 (10,000)	34 (14)	ND (-)	ND (-)
	After	300 (140)	5.6 (0.2)	2,000 (960)	ND (-)	ND (-)	ND (-)

* Concentration contents: average (standard deviation);

** N.D.: not detected.

**Table 3.** Wastewater characteristics before and after treatment with odor-reducing agents at 5 districts.

**Sites**		**BOD, mg·L^−1^**	**pH**	**SS, mg·L^−1^**	**NH_3_-N, mg·L^−1^**	**Temp., °C**
**ONE**	Before	180	7.1	120	18	10
	After	220	7.1	80	17	21
**TWO**	Before	250	7.6	80	10	10
	After	190	7.8	90	16	14
**THREE**	Before	220	8.4	130	53	9.6
	After	420	7.5	240	27	14.1
**FOUR**	Before	260	8.1	135	200	9.4
	After	290	8.3	120	170	13
**FIVE**	Before	250	6.9	170	9.4	23.5
	A fter	160	8.9	160	13	12.5
